# Beyond Deterministic Fetal Programming: Intrauterine Exposures and the Multifactorial Origins of Adiposity

**DOI:** 10.1111/obr.70133

**Published:** 2026-04-19

**Authors:** Gernot Desoye, Ursula Hiden, Evelyn Jantscher‐Krenn, Herbert Fluhr, Jonathan C. Wells, Sonja Entringer, Mireille N. M. van Poppel

**Affiliations:** ^1^ Department of Obstetrics and Gynaecology Medical University of Graz Graz Austria; ^2^ Research Unit Early Life Determinants (ELiD) Medical University of Graz Graz Austria; ^3^ Childhood Nutrition Research Centre, Population, Policy and Practice Department UCL Great Ormond Street Institute of Child Health London UK; ^4^ Charité − Universitätsmedizin Berlin, Corporate Member of Freie Universität Berlin, Humboldt‐Universität zu Berlin Institute of Medical Psychology Berlin Germany; ^5^ Department of Pediatrics, School of Medicine University of California Irvine California USA; ^6^ Department of Human Movement Science, Sport and Health University of Graz Graz Austria

**Keywords:** (epi)genetics, body composition, DoHaD, infants, neonates, pregnancy, sex

## Abstract

Excess adiposity is not a recently developed problem but has existed since at least the upper Paleolithic, allowing evolutionary selection pressures to adapt the physiology of the pregnant woman and the feto‐placental unit for maternal and fetal protection. Disturbances of the intrauterine environment, in particular metabolic derangements early in pregnancy, such as in women with diabetes and/or obesity, may lead to fetal hyperinsulinaemia. This often results in neonatal adiposity, which may in turn increase the risk for childhood adiposity. In this review, we summarize evidence on possible underlying mechanisms and factors that moderate the association between in utero exposure and childhood adiposity focusing on pregnancies in women with diabetes or obesity. We conclude that in humans causal evidence is missing for fetal programming of adiposity in the deterministic sense as often used and propose a more opportunistic framework. A variety of postnatal exposures and their interaction with the individual's genetic background contribute to the multifactorial problem of excessive adiposity, which may be further compounded by varying sensitivity to environmental perturbations depending on the developmental stage of the individual. We identify research gaps and describe future studies to generate causal evidence necessary.

**Abbreviations**
ADP: Air displacement plethysmography
BIA: bioimpedance analysis
BMI: body mass index
CpG: 5′‐cytosine‐phosphate‐guanine‐3′
DALI: Vitamin D and Lifestyle Intervention of gestational diabetes mellitus prevention
DXA: dual‐energy X‐ray absorptiometry
GDM: gestational diabetes mellitus
GWAS: Genome‐wide association studies
HAPO: Hyperglycemia and Adverse Pregnancy Outcome
IGF‐1: insulin like growth factor‐1
IGF‐2: insulin like growth factor‐2
MRI: magnetic resonance imaging
MSCs: mesenchymal stem cells
oGTT: oral glucose tolerance test
PM: particulate matter
PPAR‐γ: peroxisome‐proliferator‐activated receptor gamma
RCT: randomized controlled trial
TOBEC: total‐body electrical conductivity
TOBOGM: Treatment of Booking Gestation Diabetes Mellitus
ucMSC: umbilical cord MSCs
WHO: World Health Organization
ZNF423: zinc finger protein 423

## 1 Introduction

Obesity has been on the rise over the past decades, not only in human but, interestingly, also in feral and domesticated animals living with or around humans [1]. The current obesity epidemic in humans creates a burden for public health and calls for actions to reduce it. In 2022, about 890 million people worldwide had obesity with numbers continuously on the rise [2]. Alarmingly, there is also an increasing number of children who are obese, with a latest estimate of about 160 million children and adolescents aged 5–19 years with obesity [2, 3], and children with obesity are more likely to become obese in adulthood [4]. In order to find adequate preventive measures it is necessary to understand the etiology of obesity [5]. There is growing evidence that the first 1000 days of life, that is, the intrauterine period, including conception, and 2 years thereafter, play an important role in determining offspring obesity susceptibility and, thus, risk for becoming obese later in life [6, 7]. The term “developmental programming” has been coined to emphasize the relevance of this period [8, 9], not only for obesity risk but also risk for other associated conditions, including Type 2 diabetes, cardiovascular problems, hypertension, and metabolic syndrome. “Fetal programming” refers to the embryonic and fetal period, during which cell differentiation and extensive cell proliferation make cells and tissues particularly susceptible to environmental perturbations [10, 11, 12].

Obesity is a complex, polygenic, and heterogeneous trait as manifested in various obesity subtypes [13]. Both genetic predisposition as well as plastic, potentially amenable components may contribute to obesity [14]. In adults, genes or the environment can only explain up to 50% of phenotypic variation of complex traits such as obesity [15, 16]. A better understanding of factors contributing to the other 50% of variation is essential in the prevention and treatment of obesity. Multiple studies have demonstrated associations between early childhood body fat or body composition with health outcomes, including obesity, later in life [17, 18]. Classically, increments in fat deposition have been attributed to energy intake exceeding energy expenditure (“energy balance model”). In recent years, additional models have been proposed, and some effort was made to integrate these into holistic frameworks [19, 20, 21, 22].

Among the multiple pathways through which the mother can influence the phenotype of her offspring [23], the concept of *developmental programming* is the most prominent, as reflected by the large number of reviews published on this topic (e.g., [9, 24, 25, 26, 27, 28, 29]). These reviews predominantly address the developmental programming of obesity from specific perspectives, focus on defined mechanisms, and often integrate evidence from both human and animal—mostly rodent—studies.

However, obesity is not synonymous with adiposity (cf below), and animal models do not fully recapitulate human prenatal and postnatal exposure patterns or their effects on later‐life adiposity, which constitutes a major translational limitation. Numerous interspecies differences contribute to this gap, including reproductive strategy [30], placental characteristics [31] and evolutionary history [32]. Critically, the timing of adipogenesis differs markedly: rodents are born with almost no adipose tissue, as the majority of adipogenesis occurs postnatally during lactation [33]. In addition, differences in adipose tissue depot organization [34], adipocyte differentiation [35], their architecture [36], and progenitor cell niches [37] further distinguish adipogenesis and adiposity phenotypes between humans and rodents [38, 39].

For these reasons, we limit our discussion to human studies and focus on whether elevated adiposity—defined as excessive enlargement of fat depots, which is commonly observed in individuals with obesity and represents the primary harmful factor in obesity [38]—is determined during development in utero, that is, during the fetal period, and whether adiposity at birth tracks into infancy and childhood. This review centers on pregnancies in women with diabetes and its associated hyperglycemia or obesity, for which intergenerational, but not transgenerational, associations with adiposity of human progeny have been described in several populations [40, 41]. Because it is difficult in human populations to disentangle the effects of preconceptional exposures from those occurring during subsequent stages of pregnancy on offspring anthropometric outcomes [39], we primarily discuss exposures during pregnancy and expand this framework to include multiple exposures that may contribute to adiposity later in life. The framework presented here aims to facilitate a better understanding of adiposity as the result of complex interactions between genetic factors and the prenatal and postnatal environment.

## 2 Difference Between Obesity and Adiposity

In clinical practice, adult obesity is a categorical measure based on body weight relative to height and conventionally defined as body mass index (BMI) ≥ 30 kg/m^2^, though lower cutoffs have been proposed for Asian populations, as adverse health effects emerge at lower BMI thresholds [42]. In younger age groups, BMI standard deviation scores are used [43]. As a composite quantity of weight and height, BMI does not differentiate between tissues, and does not provide information on body composition [44]. The simplest model of body composition encompasses two compartments: lean and fat mass. Prenatal and postnatal development of both compartments are differently regulated, resulting in dynamic changes in body composition soon after birth [45, 46]. Whereas insulin is the main driver for fat mass, lean mass regulation including skeletal size is more complex and involves many factors known and unknown, including insulin [47, 48, 49]. For any given BMI, body composition can be different, not only among adults [50] but also already at birth. For instance, offspring born to pregnancies when the mothers had had type 1 diabetes, gestational diabetes mellitus (GDM) or obesity can have increased fat mass [51], even when they are in the same birth weight category (e.g., large for gestational age, appropriate for gestational age) as offspring with less fat mass from healthy mothers [52, 53]. Hence, identifying a convincing causal etiology for obesity as health outcome is difficult.

Adiposity is a term that describes the relative amount of body fat. It is a continuous measure defining fat mass, often expressed as percent relative to body weight. However, changes in % body fat do not inform about changes in the fat compartment alone, as any change may be accounted for by either a change in fat mass or lean mass or both [54]. Hence, other normalization strategies have been proposed [55], such as the lean mass index (lean body mass/height^2^) and fat mass index (body fat mass/height^2^), which have become increasingly used in epidemiological studies (e.g., references [56, 57, 58, 59, 60, 61]). Another index introduced is the relative fat mass (RFM), which correlates strongly with trunk adipose tissue levels in adults [62]. It has been suggested to use the terms “overfat” and “underfat” instead of BMI categories to indicate excessive or too little fat mass relative to lean body mass, respectively [63]. The Body Roundness Index [64, 65, 66] and the Trunk Body Mass Index [67] were also proposed as more informative measures that take body fat distribution into account. Their utility in childhood awaits assessment.

The advantage of using obesity as an outcome in exposure‐outcome analyses is that weight and height data are usually available in registry studies as well as in epidemiological studies, because both are easy to measure. However, the value of obesity, or specifically of BMI, as a health proxy has been questioned for decades as it does not adequately reflect metabolic dysregulation or adiposity [68, 69, 70, 71] and has led to new definitions of obesity [72, 73, 74]. Similarly, for neonates, variability in birthweight has been regarded as inadequate as an outcome of in utero exposures [75].

## 3 Assessment of Adiposity in Fetus and Offspring

Adiposity can be measured by a range of methods that mostly target body composition [76, 77, 78, 79, 80]. At birth, the easiest method uses calipers to measure subcutaneous fat thickness [81] usually at three predefined anatomical locations. From these measurements, total neonatal body fat can be calculated based on formulas [82], few of which take sex into account [83, 84], although skin fold thickness and fat mass differ between sexes [47]. Alternatively, Advanced Principal Component analysis of multidimensional anthropometric data in neonates can be used to extract sex‐specific skeletal size and body fat information [47]. In infants, skinfold thickness is measured at up to five body sites [85]. For conversion to total body fat estimates, two widely employed equations [86, 87] correlate only moderately with fat measurements using total‐body electrical conductivity (TOBEC) [85]. Sex‐specific anthropometric models that correlate more strongly with measured adiposity are available for the first year [82].

Bioimpedance analysis (BIA) is an easy method often used in epidemiologic field studies. However, its reliability decreases in infants or individuals with high adiposity [46] and it requires careful calibration for each study population to ensure accuracy [88, 89, 90, 91]. TOBEC measurements allow for reproducible, safe and validated estimation of fat and fat‐free mass in neonates [52, 53, 92, 93, 94, 95, 96] and infants [97]. An alternative, but expensive method to assess fat and fat‐free mass is air displacement plethysmography (ADP). It can be used from birth to 6 months [98] and from early childhood to old age [99], thus obviating the need for changing methods when studying longitudinal developmental trajectories. Other advanced and expensive methods include dual‐energy X‐ray absorptiometry (DXA), which has the downside of variable accuracy depending on tissue depth and fat mass [100, 101, 102, 103]. Isotope, that is, deuterium, dilution combined with isotope ratio mass spectrometry is an accurate method and, if used in a four component model, allows assessing not only fat but also water, protein, and mineral content [104]. Deuterium is suitable for all age groups from birth to older age [105] with reference data available in infants and children [106, 107]. Magnetic resonance imaging (MRI) has the benefit that it can also assess regional variation in body fat deposition. This highly accurate and reproducible method provides volume estimates of fat tissue, lean tissue and total body water [46, 108]. A very recent development is EchoMRI, which has been applied to neonates between 12 and 69 h after birth and offers some advantages over conventional MRI [109]. However, when compared with ADP and DXA, it underestimates fat mass in infants and children at the group level. The 3‐D optical imaging is another emerging technology that has been used in children between 5 and 17 years of age, but not yet in neonates [110].

An inherent problem with some of these methods, foremost fat measures based on skin fold thickness and BIA, is the requirement of prediction equations to estimate body components. These equations often have been validated for some populations and may not be applicable to others. Rapid body composition changes after birth, mostly because of loss of body water [111], further compound the problem of getting valid and reliable measurements in neonates [112].

For assessing total and site‐specific fetal fat in utero, MRI using different measuring modalities has been employed [113, 114, 115]. However, the high cost and the potential of motion artifacts limit the MRI approach to specific research questions and settings. Easier and less costly is conventional fetal biometry using 3‐D ultrasound to quantify abdominal circumference [116] or fetal fractional limb volume [117]. However, poor reproducibility of operator‐dependent selection of the ultrasound plane for measurement contributes to large inter‐ and intra‐observer variation. More advanced techniques use 3‐D volume scans at various anatomical locations together with offline tomographic ultrasound imaging analysis. With the resulting data, artificial intelligence‐based classifier models can be trained [116]. Fractional volumes of fetal extremities measured with 3‐D ultrasound associate with cord blood insulin and leptin [117] and neonatal fat mass [118].

In summary, although easy to use and reproducible, BMI or birthweight category as a measure of offspring health is inadequate. Body composition assessment in early life is more complicated and challenging in a clinical setting, but this may be overcome with adequate resources [119]. Data using different methods are increasingly obtained and advance our understanding of the determinants of adiposity in early life. Not all measures are suitable for all age groups, making the assessment of longitudinal development of body composition difficult. However, studies using birthweight (category), BMI or obesity as outcome will not further our understanding of the etiology of adiposity, and serious efforts are needed to include body composition measures in large‐scale longitudinal studies.

## 4 Evolution of Adiposity

Compared to other primates, humans have both higher overall levels of body fat, as well as a complex pattern of variability by age and sex [120]. This indicates that adiposity was uniquely important in human evolution, increasing the capacity of our ancestors to survive and produce offspring in a wide range of ecological conditions and in volatile environments [121, 122]. Although adipose tissue is not directly preserved in the fossil record, it is likely that the profile of hominin adiposity changed most substantively within the genus *Homo*, and in particular with the emergence of *Homo sapiens* , in association with trends to lower lean mass, increased fetal brain size and changes in female reproductive energetics [123, 124]. The changing pattern of adiposity by age reflects the substantial lengthening of maturation and lifespan in our species, while the elevated adiposity in adult females relative to males highlights its role in funding lactation [125].

During pregnancy, a typical gestational weight gain of 12.5 kg involves the accumulation of around 3.8‐kg fat, representing 152‐MJ energy stored over 9 months [126]. During the first 5 months of lactation, the costs of exclusive breastfeeding (estimated at 2.6 MJ/day) are met in part by oxidation of these fat stores (contributing around 0.7 MJ/day), and in part by elevated dietary intake [126], although these proportions vary within and between populations depending on cultural practices and food availability. However, whether breastfeeding systematically reduces post‐partum weight retention remains unclear, and may depend on contextual factors including the duration of breastfeeding [127, 128].

A systematic review of studies in healthy term breastfed infants found that variability in breastmilk carbohydrate content was associated with infant weight gain, whereas protein and fat content were not [129]; however, the quality of evidence remains poor. Maternal BMI has been associated with higher levels of leptin and insulin, and the ratio of omega‐6 to omega‐3 polyunsaturated fatty acids in breast milk [130]. Maternal obesity and gestational diabetes may alter the macronutrient composition of breastmilk, but findings to date are heterogenous [131, 132]. The potential role of breastmilk composition in transmitting metabolic effects of maternal obesity to the offspring therefore requires further research [32].

Over 1100 individual alleles have already been associated with adiposity phenotypes, most commonly with BMI but also with fat distribution as indicated by the waist‐hip ratio [133]. Although the magnitude of effect on BMI per allele is typically small, selection has likely acted on such alleles because they are associated with other physiological traits. Given the instability of environments within and across generations, humans seem to have evolved a complex genetic basis of adiposity. On the one hand, the highly polygenetic basis of adiposity reduces the exposure of individual alleles to selection. On the other hand, variability in the ‘fitness value’ of fat is accommodated through mechanisms of plasticity, depending on the endogenous characteristics and environmental conditions of the individual [134]. Our genetic architecture thus underpins reaction norms that enable fat stores to be rapidly gained and lost over time. To different degrees at different ages, fat stores can buffer dietary energy shortfalls and fund growth, immune function and reproduction, while also emitting biological signals that regulate diverse physiological processes in association with nutritional status [123]. While obesity research has focused on the potential harms of excess fat, inadequate fat is also a risk factor for mortality [135]. For example, in a high altitude setting in Ladakh, north India, low neonatal adiposity was a strong predictor of neonatal and infant mortality [136]. Body fat is thus central to a range of life‐history trade‐offs, whereby it confers a range of metabolic benefits but imposes non‐communicable disease risk costs at higher levels.

Although obesity is often presented as a relatively recent phenomenon that only emerged in the agricultural era, the upper Paleolithic archeological record provides numerous female figurines showing accurate depictions of female obesity [32], indicating that maternal overweight has been exposed to selection for many millennia. During this period, some capacity to defend against nutritional excess may have evolved [137, 138].

## 5 Development of Fetal and Neonatal Adiposity

Adipose tissue comprises at least three types of adipocytes, that is, white, brown, and beige [139]. Brown and beige adipocytes share thermogenic capacity, whereas white adipocytes have the capacity to store extra energy in the form of fat. In the human fetus, brown fat covers predominantly the cervical, thoracic and abdominal viscera [140]. Here, we focus on white adipocytes and white adipose tissue as it is strongly linked with metabolism [141]. The triglyceride content, reflected in cell volume, but not the number of white adipocytes, responds postnatally to environmental cues [142], although some trans‐differentiation between white to brown adipocytes may contribute to modest changes in white adipocyte number [143].

The first triglyceride depots in the human fetus can be measured already at around 10 weeks of gestation with a continuous increase thereafter. Activity of acetyl Co‐A carboxylase, the rate limiting enzyme for fatty acid synthesis, can be measured at 13 weeks. It is under insulin regulation, which is present in fetal adipose tissue some weeks later, likely in the developing capillary network [144, 145].

The first fat lobules, that is, groups of adipocytes enclosed by a membrane, in the fetus can be seen at around 14 to 16 weeks and continuously increase in number until around 23 weeks [146, 147]. During this period, the size of the fat lobules is linearly related to fetal crown rump length [148, 149]. Thereafter, adipose tissue growth is mainly driven by the increase in size of fat lobules, the emergence of mesenchymal cells and by the growing capillary network [149]. Importantly, development of subcutaneous adipose tissue, which makes up most of neonatal fat [101, 149], begins later than other adipose types [150] and is mostly deposited in the third trimester [151].

In normal pregnancies, the human neonate contains about 12%–16% body fat at birth, which is the highest proportion among mammals [33]. In pregnancies of women with diabetes or obesity, more fat is deposited, mostly in subcutaneous locations [51, 52, 152, 153, 154, 155, 156, 157, 158, 159, 160, 161]. Predominantly, the extent of maternal hyperglycemia underpins neonatal adiposity, independent of the underlying pathophysiological mechanism [157, 158, 162]. The specific timing and onset of the increase in fat deposition in utero is unknown. Longitudinal measurements of fetal abdominal circumference show a dynamic increase from about 20 weeks onward in women who were obese or later diagnosed with GDM [163]. At 14–16 weeks, abdominal circumference is smaller in fetuses of women with obesity or those later diagnosed with GDM. At that gestational age, fetal abdominal circumference is likely predominantly determined by fetal liver size [164] rather than by subcutaneous fat. After 30 weeks, abdominal circumference is often found to be larger in fetuses of women with GDM or obesity, to which a larger liver may contribute only in part [164]. At delivery, 63% of the variation in abdominal circumference was accounted for by abdominal fat, whereas only 3% by liver size/length [154]. The increase in abdominal circumference in the third trimester may therefore represent a rise in subcutaneous abdominal fat. However, subcutaneous fat deposition is a dynamic process and its excess may have commenced much earlier, without reaching statistical significance earlier in pregnancy in many studies.

Increases in adipose tissue can result from either an increase in adipocyte number, that is, adipocyte hyperplasia, or adipocyte lipid storage thereby increasing cell size or volume, that is, adipocyte hypertrophy. In both processes, fetal insulin plays a central role and, thus, is the main driver and regulator of fetal adiposity. Insulin can act as growth factor and metabolism regulator, and contributes to proliferation and differentiation of pre‐adipocytes to mature adipocytes and to triglyceride formation and deposition in adipocytes [147]. In the following, the mechanisms underlying neonatal adipocyte number and adipocyte lipid storage are addressed.

### 5.1 Regulation of Adipocyte Number

Subcutaneous adipocytes are the first storage sites for lipids. At birth, intra‐abdominal or visceral adipose tissue stores only small amounts of fat [101]. Only when the maximum storage capacity of subcutaneous adipocytes is reached will excess fat spill over and be stored at ectopic sites, foremost in the liver but also in the epicardium [165]. Thus, adipocyte number is an important contributor to an individual's health. Infants of 1–3 weeks of age born to women with obesity and GDM have about 70% more intra‐hepatocellular fat than those born to normal weight mothers without gestational diabetes [166], which may predispose them to fatty liver disease.

**FIGURE 1:**
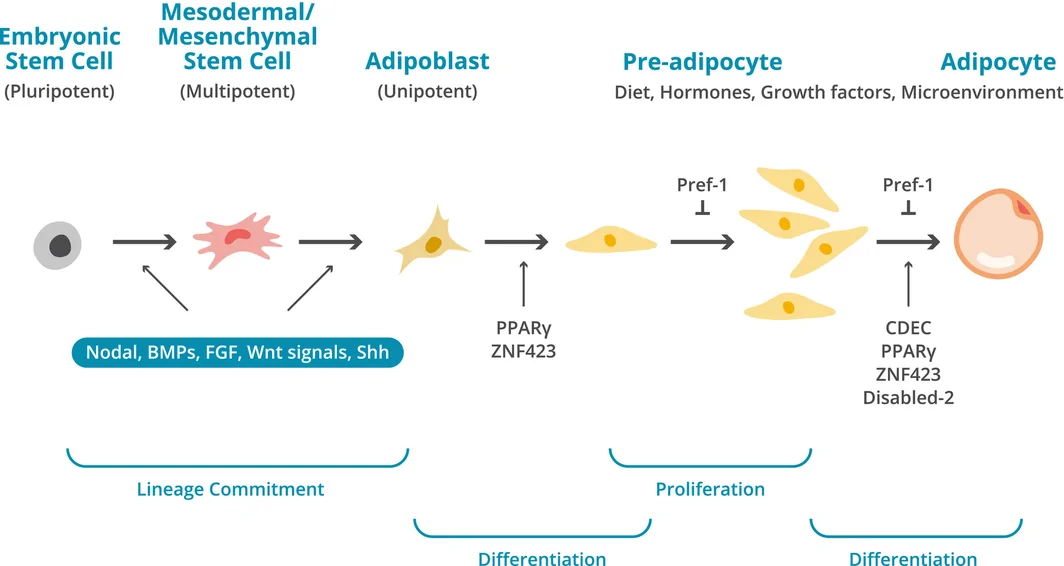
Prenatal adipogenesis and presumed regulators. Embryonic stem cells commit to the adipocyte lineage, a process governed by a complex genetic network. Subsequently, serial differentiation and proliferation steps ultimately form mature adipocytes capable of storing triacylglycerols. Multiple known and presumably unknown intrinsic factors including insulin and IGF‐1 and environmental cues act in concert to drive the various steps and hence determine adipocyte number. Redrawn from [147]. Abbreviations: BMP: bone morphogenetic protein; CDEC: Cell Death Inducing DFFA (DNA fragmentation factor‐A) Like Effector C; FGF: fibroblast growth factor; PPAR: peroxisome proliferator‐activated receptor; Pref‐1: pre‐adipocyte factor 1(also Dkk1); Shh: sonic hedgehog; Wnt (wingless‐type MMTV [Mouse Mammary Tumor Virus] Integration Site); ZNF: zinc finger transcription factor. ↑: stimulating effect; ⊥: inhibitory effect.

Details of prenatal adipogenesis and its regulators are poorly understood in humans [147, 167]. The pathway from embryonic stem cells to mesodermal/mesenchymal stem cells to the unipotent adipoblast lineage is mainly governed by a complex gene network (Figure 1). The possible interaction of the genes involved with the fetal environment has remained elusive so far. Regulators of adipoblast differentiation to pre‐adipocytes include the transcription factors peroxisome‐proliferator‐activated receptor gamma (PPAR‐*γ*) and zinc finger protein 423 (ZNF423). PPAR‐*γ* is activated by polyunsaturated fatty acids, predominantly the omega‐3 fatty acids docosahexaenoic acid and eicosapentaenoic acid, whereas COUP transcription factor 2 activity may determine ZNF423, a multi‐zinc finger transcription factor. These two transcription factors, PPARy and ZNF423, are also involved in regulating pre‐adipocyte proliferation and differentiation to adipocytes. An additional role is played by circulating factors, including pre‐adipocyte factor, insulin, and insulin like growth factor‐1 (IGF‐1), all found in the fetal or neonatal circulation [147].

Since ZNF423 is epigenetically regulated, it is susceptible to environmental perturbation and might potentially contribute to long‐term consequences such as increased adipogenesis in the postnatal period [168]. In combination with increased insulin levels, it may be one of the reasons underlying the higher adipocyte number in neonates born to women with GDM compared with those born to women without GDM [153, 169], and in toddlers with obesity at 2 years of age [142]. The higher adipocyte number in individuals with obesity was found throughout the life course in a cross‐sectional analysis [142].

**FIGURE 2:**
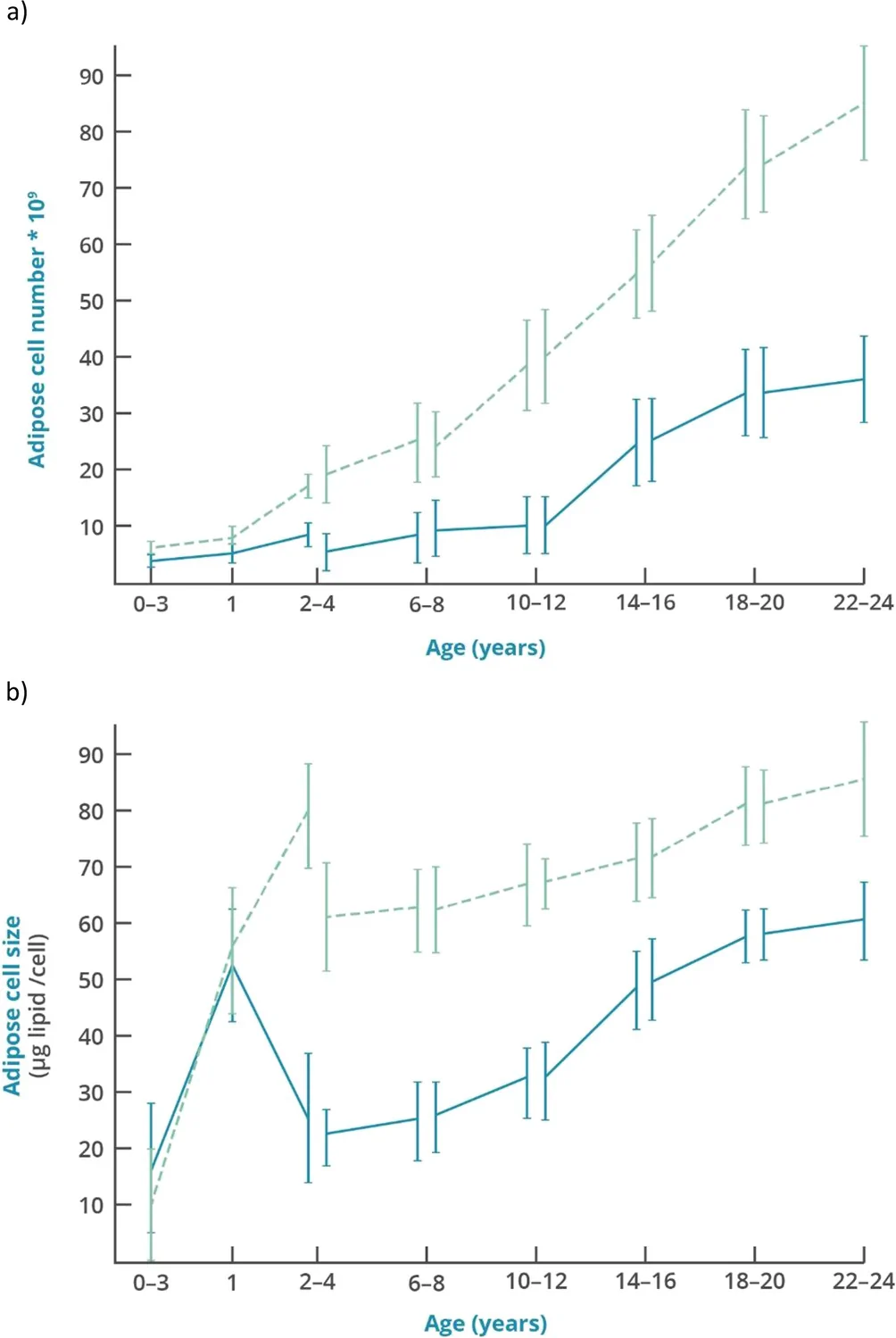
Adipocyte development from infancy to adulthood. Longitudinal changes in (a) adipocyte cell number (mean ± SEM) and (b) adipocyte cell size (mean ± SEM) during the period from 4 months to 24 years in individuals with (green) and without obesity (blue). Modified from [170].

This is in line with findings of a longitudinal study [170]. From 6 months to 1 year, increments in fat depots were mostly accounted for by an increase in cell size and not cell number [170]. However, at the ages of 4 months and 1 and 2 years, offspring who had obesity later in life had more adipocytes, although this difference was significant only at the age of 2 years [170] (Figure 2). There was a significant increment in cell number between 1 and 2 years of age in both children who developed obesity later in life and those who remained normal weight. After 2 years of age, adipocyte numbers did not further increase in individuals with normal weight, whereas in individuals with obesity, their adipocyte number continued to increase until 24 years of age [170]. This finding at one body site (buttocks) of offspring without obesity is at variance from a continuous increase in adipocyte number across different body sites [151]. Thus, adipocytes may proliferate in a site‐specific manner. Offspring sex did not significantly influence cellularity [151, 170].

Our understanding of the complex, multi‐step mechanisms of adipogenesis in human fetuses is still incomplete. However, existing evidence suggests that pregnancies affected by maternal overnutrition, such as diabetes or obesity, create an altered fetal environment, characterized by elevated levels of insulin and IGF‐1. These changes can affect prenatal adipogenesis, potentially increasing the number of adipocytes in the fetus and neonate.

### 5.2 Regulation of Adipocyte Lipid Storage

**FIGURE 3:**
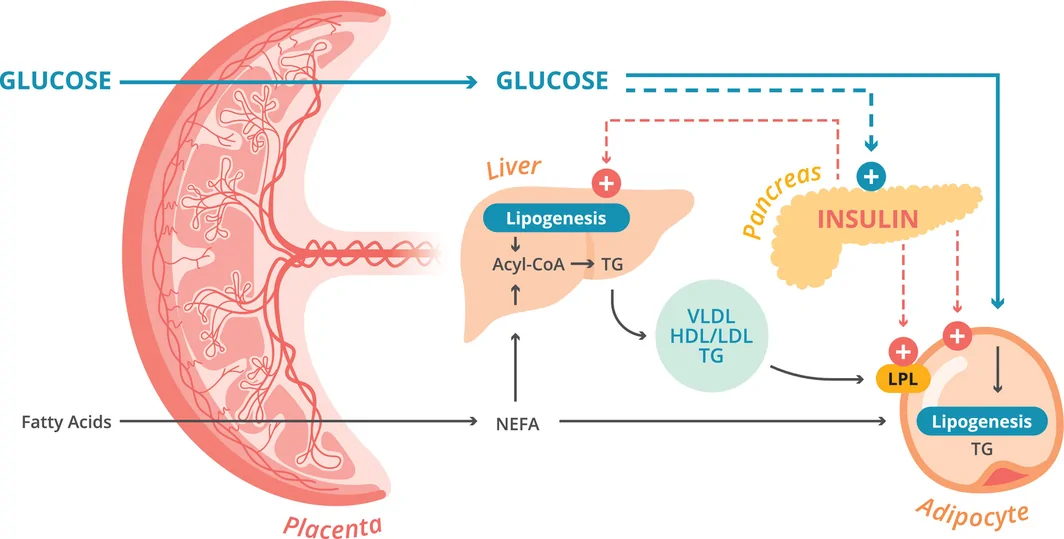
Fetal lipogenesis and the effects of the intrauterine environment associated with diabetes or obesity of pregnant women. Fetal liver and adipocytes collaborate in fatty acid and triglyceride (TG) synthesis. Glucose is the main precursor for fatty acid synthesis in the liver and adipocytes, which is stimulated by insulin in both tissues. Fetal concentrations of both, glucose and insulin, are elevated in pregnancies with diabetes or obesity. Hepatic TG can be formed either from circulating non‐esterified fatty acids (NEFA) or de novo generated Acyl‐Co. TG are then packaged into lipoproteins (VLDL, LDL, and HDL), which are released into the fetal circulation. In the adipocytes, due to the action of lipoprotein lipase (LPL), TG‐rich lipoproteins are partially hydrolyzed into NEFA. These are taken up by adipocytes to contribute to the intracellular fatty acid pool for subsequent TG synthesis. Modified from [147]. Blue lines indicate glucose effects, red lines indicate insulin effects.

In conditions of maternal hyperglycaemia that result in fetal hyperglycaemia, the fetal pancreas is stimulated to produce more insulin. Fetal hyperinsulinaemia then stimulates lipogenesis and triglyceride synthesis in fetal adipocytes (hypertrophy). At the end of pregnancy, neonatal, that is, cord blood, insulin levels positively correlate with neonatal fat mass [171]. This correlation appears stronger in male neonates [172], which could reflect the higher insulin sensitivity in male than in female neonates [173, 174, 175]. Insulin may not be the only fetal hormone influencing adiposity, as IGF‐1 also correlates positively with adiposity at birth [176]. However, IGF‐1 may just mediate the insulin effect through insulin's induction of IGF‐1 synthesis (Figure 3). This insulin‐IGF‐1 effect may only operate postnatally [177, 178], when the permissive effect of growth hormone on IGF‐1 becomes fully activated [179].

The onset of fetal hyperinsulinaemia is unclear and certainly varies between individuals and metabolic conditions of the pregnant women. Insulin can first be detected in the fetal circulation and amniotic fluid at around 11–14 weeks. At that time, fetal triglycerides are present in measurable quantities [144]. Insulin levels at 14–19 weeks are related to neonatal risk of being born large for gestational age [180], which may be an indication of excessive adiposity in many of these neonates. It is pertinent that earlier in pregnancy, that is, in 9–10 weeks, maternal glycaemia levels correlate with offspring risk of being born large for gestational age [181]. Although at this early stage in gestation the fetal pancreas may not yet be capable of releasing insulin, sustained hyperglycemia may accelerate development of beta cells and their maturation of the glucose‐insulin stimulus secretion coupling mechanism [182, 183, 184]. Thus, effects of fetal hyperinsulinaemia on development of fat depots and fat deposition (cf. below) may be present already in the second trimester of pregnancy although this notion awaits further support.

In neonates, undifferentiated mesenchymal stem cells (MSC) isolated from cord blood of mothers with obesity contained more ZNF423 protein than those from lean mothers [185]. After in vitro differentiation, they were larger and stored more fat when derived from mothers with obesity compared to lean mothers [185, 186]. Notably, the fat, that is, triglyceride, content of the MSC‐derived adipocytes associated with adiposity assessed as % fat mass of children at 4–6 years of age [187].

In summary, increased adiposity of neonates of mothers with obesity might be due to a combination of increased adipocyte number and size. However, is neonatal adiposity, as a reflection of the in utero exposure, predisposing offspring for adiposity later in life?

## 6 Does Excessive Neonatal Adiposity Track Into Childhood?

In an early, small scale study neonatal adiposity linearly correlated with adiposity at the age of 9 years, whereas birth weight did not correlate with weight at that age [188]. Most, but not all, studies in larger cohorts have also found associations between fat mass at birth and at various ages in infancy/childhood (reviewed in [17]).

Greater adiposity at birth predicted greater adiposity at ~5 months, independent of infant feeding and rapid infant growth in the Healthy Start Study (*n* = 640) [189]. Neonatal fat free mass was positively associated with fat free mass, height, fat free mass index and fat mass at 3 years, and positive trends were seen between neonatal fat mass and fat mass and fat free mass at 3 years (*n* = 103) [190]. In an Ethiopian birth cohort (*n* = 353), neonatal fat mass was associated with more subcutaneous adipose tissue and fat mass index at the age of 10 years [191]. The largest cohort to date is the follow up study to the worldwide multi‐ethnic Hyperglycemia and Adverse Pregnancy Outcome (HAPO) cohort [192], which included more than 4800 mother‐offspring pairs. Adiposity of children at the average age of 11.4 years was associated with neonatal estimated fat mass, an association that was stronger when childhood adiposity was quantified by ADP (total body fat) instead of by sum of skinfold (subcutaneous fat) [193]. No sex differences in this association were found, but neonatal fat mass was not calculated by a sex‐specific equation and, thus, potential influences of sex may have been missed. Unfortunately, in this cohort no information on fat mass accretion in infancy or early childhood was available.

Not only adiposity at birth, but also the dynamics of infant fat accretion may play a role in childhood adiposity. An Ethiopian study found that not fat mass at birth, but fat mass accretion between 3 and 6 months, was associated with fat mass at 5 years [194]. Dynamics in growth (often based on weight changes, unspecified by tissue type) are also related to childhood adiposity. In a Danish study, both accelerated fetal and infant growth were independently related to overweight at 5–9 years of age [195]. This independent association of the two periods (fetal and infant) of growth has also been shown in a large (*n* = 6464) Dutch study [56]. There, fetal and infant growth acceleration were associated with higher abdominal fat mass at 6 years of age, whereas fetal growth deceleration and infant growth acceleration had the strongest association with android/gynoid fat mass ratio, a marker of waist/hip fat distribution [102]. This pattern found in Western countries might not be universal, since in low‐to‐middle income countries, accelerated fetal growth (assessed as high birthweight) and accelerated infant growth were associated with more lean mass and not more fat mass at 9 years of age [196] or in adulthood [197].

In summary, evidence suggests that having excessive fat at birth puts the offspring at risk for excessive fat also in infancy and childhood. As a consequence, attempts to prevent or reduce neonatal and infant adiposity have to commence during pregnancy, ideally as early as possible [198].

## 7 Can Excessive Neonatal Adiposity Be Prevented?

**FIGURE 4:**
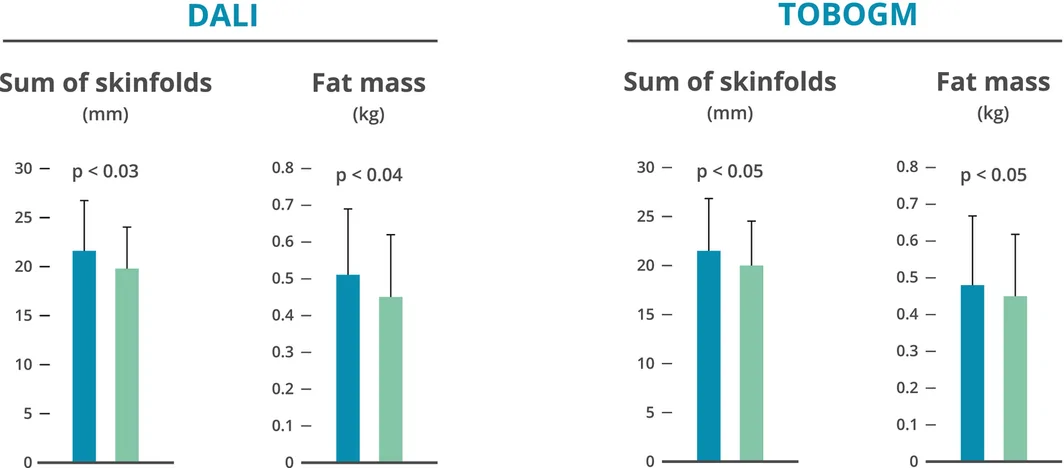
Neonatal adiposity can be reduced by lifestyle intervention in pregnant women with overweight or obesity (DALI study) or by pharmacologic intervention in women diagnosed with early GDM (TOBOGM study). A combination of healthy eating and enhanced physical activity beginning at 16 weeks in the DALI randomized controlled trial [200, 201] reduced (a) total sum of skinfolds measured at four anatomical sites and (b) estimated fat mass [202]. In the TOBOGM randomized controlled trial, neonates of pregnant women with early GDM (diagnosed at a mean of 15 weeks) receiving early GDM management had less (c) total sum of skinfolds measured at four anatomical sites and (d) estimated fat mass than those in the control group receiving standard care. The intervention effect was more pronounced when GDM was diagnosed before 15 weeks [203]. Blue bars: control group; green bars: intervention group.

Although there are not many prenatal intervention studies with neonatal fat mass or body composition as outcome, there are some promising indications that neonatal adiposity can be prevented. In a small randomized controlled trial (RCT) with 24 women, an exercise intervention from 16 weeks gestation to delivery decreased lipid content in mesenchymal stem cells and reduced infant body fat percentage [199]. In the Vitamin D and Lifestyle Intervention of GDM prevention (DALI) trial, a RCT in 436 women with BMI ≥ 29 kg/m^2^ [200], a lifestyle intervention combining healthy eating and physical activity beginning on average at 15 weeks gestation, resulted in about 9% less neonatal body fat mass (Figure 4) [202]. This represents proof of principle that excessive adiposity, which is often seen in neonates of women with obesity, can be reduced [202]. Interestingly, this intervention did not have an effect on maternal glycaemia at 24–28 weeks suggesting that the prevention effect, likely by reducing fetal insulin levels, might have occurred earlier in pregnancy. Once fetal hyperinsulinaemia is established, maternal to fetal glucose transfer is partly autonomous of maternal glucose levels, because fetal insulin steepens the glucose concentration gradient between mother and fetus [204]. This implies that interventions to reduce or prevent excessive neonatal adiposity have to begin early in pregnancy [198]. This notion was tentatively proposed already about 40 years ago, after the second trimester of pregnancy had been identified as the key period for adipose tissue development [148, 149].

While not the primary outcome, the above proposal was tested in the recent Treatment of Booking Gestation Diabetes Mellitus (TOBOGM) RCT [203]. Pregnant women with early GDM diagnosed by an oral glucose tolerance test (oGTT) using world health organization (WHO) criteria were randomized at a mean of 15 weeks into a control group and an intervention group which received early GDM management. Neonates in the intervention group had less fat mass than those in the control group (Figure 4). The intervention effect was more pronounced when the oGTT was conducted before 15 weeks. In light of these collective results, we recently proposed to initiate a discussion about the timing of GDM screening [205] with the ultimate aim to reduce neonatal fat and other adverse outcomes as compared to the current timing of GDM diagnosis. Unfortunately, no follow‐up data are available in the DALI or TOBOGM cohorts as yet to assess whether the intervention effects were sustainable. However, the association between neonatal and infant and childhood adiposity described above may suggest such an effect.

Treatment of mild GDM diagnosed at 24–28 weeks resulted in reduced neonatal adiposity [206, 207, 208], mostly in male offspring [208]. The treatment also resulted in fewer male neonates with cord C‐peptide > 95 percentile. At follow up (age 5–10 years) [208] the treatment of GDM was associated with lower BMI z‐score in female offspring born with high neonatal adiposity, but not in male offspring. Unfortunately, no data on adiposity in childhood were presented, only height, weight, and waist circumference.

An increasing number of women become pregnant after having undergone bariatric surgery. Compared to early‐pregnancy BMI‐matched women, fetuses [209] and neonates of women with a history of bariatric surgery had less fat and fat mass [210, 211]. However, findings on childhood adiposity after bariatric surgery are inconsistent [212], and results might depend on study design, the age of the children at follow up, and the comparison group used.

Overall, literature indicates that prenatal interventions might be able to reduce neonatal adiposity, and might even have a sustainable effect in childhood. However, some (sub)groups (e.g., based on gender and genes; cf. below) might benefit from prenatal interventions more than others, or might be at higher risk of developing childhood adiposity.

## 8 Sex Differences in the Development of Adiposity

First of all, the amount and distribution of fat is different between males and females [213]. Based on skin fold measurements [214, 215, 216, 217, 218], females have more subcutaneous fat at birth than male neonates. Total fat mass relative to weight, measured with ADP [219, 220] or MRI [221], is also higher in female neonates at or shortly after birth. Superficial and deep as well as internal abdominal adipose tissue quantified by MRI within 2 weeks of birth are associated with the cord blood lipidome, also in a sexually dimorphic manner [222]. After birth, the rate of increase in fat free mass was lower in females, without a sex difference in the rate of increase in fat mass, resulting in a further increase in % fat mass in females at 5 months of age compared to males. At the age of 2 years, girls had more total subcutaneous fat than boys, with the central deposition mainly driving the difference [223].

However, not only fat mass and fat distribution are different, also associations with possible underpinning factors might differ between sexes, although findings are inconsistent. A study showed that maternal fasting glucose associated with adiposity in male, but not female neonates (*n* = 84) [224], whereas another study found stronger associations in female neonates (*n* = 305) [225]. This association between maternal fasting glucose and subcutaneous fat persisted in girls 4.5 years old (*n* = 273) [225]. In children aged 2–6 years, maternal obesity was associated with higher body fat in boys, but not in girls (*n* = 325) [226]. In contrast, maternal adiposity associated with fat mass accretion in the first 2 years of life in female, but not male offspring (*n* = 224) [227]. The association of maternal pre‐pregnancy BMI with offspring fat mass at 4 years of age was also more pronounced in girls than boys (*n* = 618) in one study [228], different from another study (*n* = 511), in which the association of maternal BMI at time of offspring birth with offspring fat mass index at the age range of 5–21 years was independent of gender. In that study, it was the paternal BMI that showed gender‐dependent association with fat mass index only in boys [229]. Fat mass trajectories from birth to 12 months were unaffected by gender although GDM influenced trajectory class membership [230]. These inconsistent findings may indicate that many sex and gender differences in associations between risk factors and outcomes in offspring are spurious and may be context dependent. We need larger studies and systematic reviews assessing sex and gender differences in the programming of adiposity, which will hopefully further our understanding of the influence of context (Table 1).

**TABLE 1:** Overview of sex and gender differences mentioned in this review.

Sex differences in outcomes		
Outcome	Timing	Sex difference
Fat mass	Birth	Females ↑ [214, 215, 216, 217, 218, 219, 220, 221]
	Infancy	Females ↑ [223]
	Childhood	Females ↑ [120]
Adipocyte number	Infancy/early childhood	No sex difference [151, 170]
Cord blood leptin	Birth	Females ↑ [231, 232]
Insulin sensitivity	Birth	Females ↓ [173, 174, 175]

Sex differences in associations between exposures and outcomes		
Exposure	Outcome	Sex difference
Neonatal adiposity	Childhood adiposity	No sex difference [193]
Cord blood lipidome	Internal abdominal adipose tissue at 2 weeks	Sex difference in multiple lipid species [222]
Maternal glucose/GDM	Neonatal adiposity	Females only [225] Males only [224]
	Childhood adiposity	Females only [225]
	Fat mass trajectory in first year	No sex difference [230]
	Cord blood methylation	Sex difference in multiple CpGs [233]
Maternal adiposity	Fat mass accretion rate	Females higher rate [227]
Maternal obesity/BMI	Fat mass in Infancy/childhood	Males only [226] Females only [228] No sex difference [229]

Studies on sex differences in intervention effects on offspring adiposity are mostly lacking. As described earlier, treatment of mild GDM was associated with lower BMI z‐score in female offspring born with high neonatal adiposity, but not in male offspring [208], but no information on adiposity in childhood was available.

## 9 Genetic Contribution to Adiposity

Since the Fat Mass And Obesity Associated (FTO) gene was the first locus found with a robust association with BMI, large genome‐wide association studies (GWAS) have been conducted to identify common genetic variants for BMI and waist‐hip ratio, a proxy for fat distribution [234, 235]. A GWAS meta‐analysis identified 941 BMI‐associated loci explaining about 6% of the BMI variance [236]. A large meta‐analysis found 203 loci associated with waist–hip ratio in women explaining 4% of the variance and 79 loci in men explaining 0.3% of the variance [237]. More informative adiposity traits have rarely been studied [238, 239, 240]. Some genetic loci are fat depot‐specific [235, 241, 242, 243] adding to the complexity of the field.

While these studies in adults have bolstered the concept of a genetic contribution to body fatness, gene variants related to fetal or neonatal adiposity have remained largely under‐researched. A recent GWAS on birth weight identified three rare variants in genes involved in adipose tissue regulation: PPARy—a key regulator of fetal adipose tissue development—and inhibin‐*β*‐subunit E and activin A receptor, both members of the transforming growth factor‐β family, and related to body fat distribution in adults [244]. In a GWAS of children, 11 gene variants were associated with body fat percentage and 19 loci associated with body fatness at age 5 and 10 years, respectively [245, 246, 247]. However, the low heritability score (0.156) for childhood fatness suggests that most variation in childhood body fatness is not genetic in origin [246].

This has prompted the hypothesis that gene–environment interactions may contribute to phenotypic variation more strongly than genotype itself [248]. Most relevant studies on how genotype moderates environmental effects on adiposity traits were conducted outside of pregnancy, and studies on gene‐intrauterine environment interactions in humans are scarce.

Prenatal intervention effects may not only be sex‐specific, but may also depend on the genotype of pregnant women and the fetus. An effect of a prenatal healthy eating and physical activity intervention on cord blood c‐peptide as a proxy for insulin was only found in women homozygous for the G allele of the melatonin receptor 1B (MTNR1B) gene, but not in women with another genotype [249]. Effects of fetal genotype have not been explored yet, despite their potential importance.

Having established that an intrauterine environment as in maternal obesity or diabetes is associated with increased neonatal adiposity, which in turn is related to increased risk of childhood adiposity, this leaves the question about causality: Does the fetal environment directly influence susceptibility to the development of adiposity in childhood?

## 10 Does the Fetal Environment “Program” Childhood Adiposity?

Different periods may lead to childhood adiposity: periconception, pregnancy and infancy, with neonatal adiposity being the outcome of the first two, and the starting point of the latter. The periconception, prenatal and postnatal environments differ in their complexity that may have selected for different adaptive, that is, protective, responses during evolution [250].

The fetal programming concept posits that specific events or environmental conditions during critical periods of fetal development lead to permanent effects on the fetus and offspring and even extend into adulthood. The first evidence that an adverse fetal environment such as that associated with diabetes in pregnant women has long‐term effects on the neonate and infant came from seminal, but underappreciated studies, about 50 years ago. Offspring born to mothers with diabetes during pregnancy showed hyperinsulinaemia and diminished insulin response to glucose load at 1–8 years of age [251]. Since then, multiple studies have confirmed associations of the intrauterine milieu, in particular of maternal hyperglycaemia and fetal hyperinsulinaemia, with adverse childhood conditions [193, 225]. As insulin is the main driver of fat formation and deposition, one can assume such an association also with childhood adiposity.

The association studies of in utero environment with childhood conditions were taken as supportive evidence. Although, in principle, exposure‐outcome associations do not allow conclusions on directionality and may also reflect reverse causation, in the present instance of in utero exposure linking with long term consequences, the timeline clearly excludes reverse causation. However, these studies by no means provide causal evidence and carry a high risk of residual confounding. It is extremely difficult in humans, if not impossible, to separate intrauterine (prenatal) from extrauterine (postnatal) effects on outcomes in childhood or adulthood. An attempt was made using Mendelian randomization, but no strong causal relationship between increased maternal BMI and greater fat mass index in offspring from ages 7 to 18 years was found [252].

Given the difficulty of establishing causality with intrauterine exposures, it was proposed to discard the term “programming” [253], to clarify that early exposures may shape susceptibility to disease, rather than disease itself. The use of “environmental induction” of phenotype was suggested [254] instead. It implies that the intrauterine environment provides a prediction about the world the offspring will inhabit postnatally and, thus, the offspring will make adaptive choices to this predicted future environment [254]. Another theory suggests that it is rather the condition of the mother (as a reflection of her life experiences) that influences fetal adaptations [253]. In both alternative concepts, the deterministic ‘programming’ concept, though attractive, is replaced by an “opportunistic” view, where disease risk develops cumulatively through successive exposures.

In particular, exposures in the postnatal period need careful consideration. Among postnatal exposures, shared environment and lifestyle (e.g., diet, physical activity, and sleep) between mother and infant [255] may make in utero diabetes/obesity exposure‐outcome associations spurious. In addition, breastfeeding has a moderating effect on these associations, suggesting some protection against later adiposity [57, 223, 256, 257] or in the case of mothers with diabetes, rather increasing the risk of later overweight [258]. The content of infant formula may also influence fat development: infant formula with lower protein content resulted in lower fat mass in children aged 2 to 6 years [259]. Weight gain in the first week after birth plays an important role in the association of formula‐feeding with obesity risk later in life [260]. Furthermore, introduction of complementary food increases rates for accrual of abdominal and hepatic fat [261] with timing of complementary feeding modifying adiposity risk [262].

Infant gut microbiota may also play a role in shaping the degree of infant adiposity [263]. Indirect evidence suggests some protective role of gut microbiota composition in the first year of life reducing adiposity risk at 15–40 months of age [264]. However, a large longitudinal study found no consistent association between early life microbiota and DXA‐measured body composition at 6 years [265]. A potential contributing role of microbiota, if any, can involve short chain fatty acids, produced by anaerobic gut bacteria, through PPARy activation [266, 267] influencing adipogenesis, or through modification of the gut‐brain axis [268].

The brain is one of the master regulators of organ physiology [269]. In rat fetuses, higher brain regions, in particular feeding/satiety centers in the hypothalamus are altered by prenatal hyperinsulinaemia [270]. In rodents, epigenetic malprogramming of the hypothalamic insulin receptor leading to insulin resistance of arcuate neurons during critical periods of brain organization and the development of hypothalamic neurocircuits may underpin these hypothalamic changes [271, 272, 273, 274, 275]. There is circumstantial evidence also in humans supporting the role of perinatal hypothalamic alterations which may contribute to postnatal feeding effects on later adiposity [276, 277]. Functional alterations found in other brain regions in the fetus, when metabolism of the pregnant women is altered such as in GDM, may reflect central insulin resistance [278, 279, 280] in the neonates, especially in female neonates [173, 174, 175, 281], and also in children of mothers with diabetes [282]. Central insulin resistance is linked to adiposity and body fat distribution [283] through pathways controlling feeding behavior and energy metabolism [284]. Its potential persistence into childhood may be a strong contributing factor to adiposity and obesity [285] but can be alleviated or reversed by physical activity [286].

GDM, and presumably, pregestational diabetes and obesity also affect the fetal endocrine pancreas by increasing the volume density of endocrine tissue in the pancreas and the percentage of *β*‐cells [287, 288, 289], which is regarded as an adaptive response to prolonged exposure to insulin secretagogues. The specific mechanisms underpinning enhanced *β*‐cell proliferation are unknown. Insulin is a candidate at least in animal models either acting directly in an autocrine manner mediated through *β*‐cell insulin receptors [290] or indirectly through insulin‐induced stimulation of IGF‐1 levels. This notion has been challenged [291] and a variety of alternative and novel factors [292, 293, 294] have been introduced including neuronal hypothalamic signals [295]. Regardless of its mechanistic cause(s), increased *β*‐cell number enables fetus and infant to respond to external cues, contributing to long term consequences associated with an adverse intrauterine environment.

Changes in the brain and gut‐brain axis and other unknown factors do not act in isolation, but rather in concert, forming networks that interact with the genetic background of the individuals [235, 242, 248]. These multiple and complex determinants of early life developmental plasticity [296] make precision medicine approaches for prevention of childhood adiposity enormously challenging.

Except for one previously mentioned study that did not find a strong causal relationship between maternal BMI and offspring fat mass index [252], no Mendelian randomization studies, which can provide more causal evidence, have been published for adiposity. Recent GWAS have identified several loci that could be used in those studies [245, 246, 247, 297]. The strongest evidence would come from intervention studies in pregnancy that reduce both neonatal adiposity and also childhood adiposity. We still have to await these studies.

Based on the currently available information in humans, we cannot distinguish between the influences of prenatal and postnatal exposures and thus cannot establish a causal relationship between the fetal environment and childhood adiposity. Therefore, the suggestion of a fetal “program” leading to adiposity in humans still appears premature.

## 11 The Potential Role of Epigenetics in Programming

Epigenetics refers to changes in gene function that can be mitotically and/or meiotically heritable and reversible, but are not caused by alterations in the DNA sequence. These changes involve DNA methylation at CpGs (cytosine–phosphate–guanine sites), chromatin proteins, and non‐coding RNAs [298]. Most epigenetic modifications alter the accessibility of DNA to the transcription machinery, thereby regulating gene expression [299, 300, 301], and play a central role in cell differentiation and developmental processes from the early embryonic period onward [300, 302]. Because of its inheritance, epigenetics has become increasingly recognized as a fundamental genome‐independent process underlying fetal programming through paternal environmental exposures and later disease risk [303, 304, 305, 306]. However, establishing causality between epigenetic variation and phenotypes, such as adiposity, has remained difficult [307].

Studying epigenetics in human pregnancies is complex and difficult because of the long time period required to assess effects of maternal exposure, leading to epigenetic changes in the fetus, on offspring outcomes and long‐term health risks. Poor accessibility of relevant biological samples further contributes to the difficulty. Therefore, human studies have focussed on tissues or cells that can be obtained non‐invasively or minimally invasively, such as placenta, umbilical cord, blood cells from umbilical cord vein and peripheral blood, cord blood free DNA [308] and, very recently, also in offspring buccal swaps [309, 310]. However, these are all complex tissues, and disease‐associated epigenetic changes may reflect altered cellular composition rather than cell‐type specific epigenomic modifications [311, 312, 313]. Although deconvolution methods using reference data sets have been described, these reference data sets do not include those from different maternal exposures [314]. Thus, the bulk analyses of tissue or a cell mixture do not provide mechanistic insights [315] and their value is limited to describing phenotypic variation [307] or assessing their use as potential biomarkers of outcome, for example, to predict adiposity risk in offspring [316].

Epigenomic changes were either found in genome‐wide analyses or differentially methylated regions [317] using array techniques or in pre‐specified target genes using pyrosequencing. Most studies interrogated DNA methylation, with only very few also testing hydroxymethylation of DNA [318].

### 11.1 Epigenetic Studies Using Umbilical Cord Blood

Umbilical cord blood, representing a fetal tissue, is easily accessible. Several, but not all [319], studies have identified DNA methylation changes in bulk cord blood cells associated with maternal BMI [233, 320, 321, 322, 323, 324], maternal lipids [325], or maternal glucose metabolism parameters or GDM [317, 323, 324, 326, 327, 328, 329, 330, 331]. However, it is unclear whether the associations reflect the true exposure effect under study or are the result of variations in dietary patterns, because nutrients, especially micronutrients of the 1‐carbon metabolism, may change cord blood DNA methylation [318, 332], an effect that can be independent of above exposures [332]. Moreover, fetal sex can also influence cord blood methylation changes associated with exposure (e.g., BMI [233]).

### 11.2 Epigenetic Studies Using Umbilical Cord Stem Cells

Epigenetic effects play a central role in cell differentiation, also influencing adipogenesis [333]. Lineage commitment by differentiation of mesenchymal stem cells (MSCs) into adipocytes [334] is an early event in this process and may influence adipocyte function. Indeed, outside pregnancy, DNA methylation of adipose tissue is altered in human obesity and diabetes with consequences for its function [334, 335, 336].

Outside pregnancy, progress has been made in our understanding of the significance of MSC DNA methylation from various tissue sources for terminal adipocyte differentiation and adipogenesis [337, 338] by, among others, knock‐down experiments of the DNA demethylase ALKBH1 [339]. While the epigenetic pathways involving imprinted mesoderm‐specific transcript (MEST), associated with adipocyte size [340, 341], have been identified, little is known about the role of epigenetic mechanisms governing fetal and neonatal adipogenesis. Several human studies have used umbilical cord MSCs (ucMSC) as a proxy for fetal adipocyte precursors and, after in vitro adipogenesis, as a proxy for neonatal adipocytes. In fact, ucMSCs from mothers with obesity exhibit a greater potential for adipogenesis [186] and also show signs of adipocyte hypertrophy [185]. Fat content in ucMSC‐derived adipocytes is associated with childhood adiposity and fasting glucose levels [187]. A key determinant in this process is maternal obesity, which influences not only the infant's fat mass at birth but also ucMSCs' metabolism [342, 343, 344] and the DNA methylation of key genes involved in fatty acid oxidation [343].

### 11.3 Can Epigenetic Changes Be Used for Predicting Future Risk of Obesity?

Epidemiological studies have linked intrauterine conditions to epigenetic changes in both children and adults. Adult offspring of pregnant women exposed to the Dutch winter famine of 1944/45 in early pregnancy showed not only an unfavorable metabolic profile, including higher BMI, but also differences in peripheral blood methylation of several genes, including the insulin receptor gene, which is centrally involved in metabolism [345, 346]. Interestingly, higher birth weight was also associated with insulin receptor methylation changes in adult offspring. An unbalanced maternal diet during pregnancy, with excess red meat and few carbohydrates, was associated with DNA methylation changes of the glucocorticoid receptor, glucocorticoid‐converting enzyme 11*β*‐hydroxysteroid dehydrogenase type 2, and the insulin like growth factor‐2 (IGF‐2) regulator H19 in peripheral blood of adult offspring, all of which were associated with increased adiposity [347]. The potential role of the IGF‐2 system in programming offspring adiposity was further highlighted by the association between IGF‐2/H19 methylation in umbilical cord blood leukocytes at birth and the risk of overweight/obesity at 1 year of age [348]. Another study found a correlation of RXRA (retinoid X receptor‐*α*) and NOS3 (encoding endothelial nitric oxide synthase) DNA methylation in cord blood with fat mass at 9 years of age [349]. Both prospective studies support the direct link between epigenetic programming during pregnancy resulting from maternal overweight/obesity with offspring adiposity later in life. However, causality still awaits demonstration.

### 11.4 Interventions to Prevent Epigenetic Programming

Although not unchallenged [307, 315, 316, 350], the basic hypothesis posits epigenetic changes as causal or on the mediating pathway for fetal programming of adiposity. Hence, targeted intervention or prevention strategies during and before pregnancy may be developed that in the short term prevent the epigenetic changes that are associated with neonatal and subsequent childhood adiposity. In fact, epigenetic changes, especially changes in DNA methylation, have been proposed as one of the underlying molecular mechanisms amenable to lifestyle interventions during pregnancy [351]. Weight loss improves systemic insulin sensitivity [352] and shows early beneficial effects on subcutaneous adipose tissue [353]. However, adipocytes retain an epigenetic memory of prior adiposity [336], which may undermine the sustained success of interventions. Moreover, lifestyle interventions during pregnancy that modulate the epigenetic changes in cord blood associated with maternal overweight/obesity or diabetes [354], could test the potential of DNA methylation detection in cord blood as a biomarker to predict future adiposity risk.

## 12 Does the Placenta Play a Role in Programming?

The placenta is a fetal organ with a wide range of functions to sustain growth and development of the fetus. It facilitates maternal cardiovascular and metabolic adaptation to pregnancy by releasing signals into the maternal circulation and responds to maternal changes, thus forming feedforward‐feedback loops. These may play a role in pregnancies with diabetes or obesity of the mother [355, 356]. Owing to its position, that is, interposed between the maternal and fetal circulations, the placenta mediates the maternal exposome to the fetus [357]. Hence, any changes in the maternal circulation may affect the fetus including its body composition. In diabetes and obesity, hyperglycaemia of the pregnant woman leads to hyperglycaemia in the fetus with ensuing fetal hyperinsulinaemia [155, 355, 358], the main driver for triglyceride deposition in fetal white adipose tissue [147]. Maternal‐to‐fetal glucose flux is mainly dictated by the concentration gradient between both circulations with some potential for a minor contribution of the placenta itself in situations of profoundly disturbed maternal metabolism [204, 359]. The very limited trans‐placental transfer of maternal fatty acids to the fetus makes their contribution to excessive triglyceride formation and fat deposition unlikely. In maternal hyperglycaemia, abundant glucose in the fetus serves as precursor for lipogenesis, obviating the need for fatty acids as building blocks for fetal triglycerides [147].

Whether the placenta itself plays a causal role in changing fetal susceptibility to postnatal environmental challenges that ultimately lead to infant or childhood adiposity is unknown. Principle mechanisms would include soluble factors such as metabolites, cytokines, growth factors, hormones, or particles such as extracellular vesicles that are released from the placenta into the fetal circulation. There they need to modify fetal systems that are involved in development of postnatal adiposity. Some statistical associations between placental changes and childhood adiposity have been found (e.g., [360, 361, 362, 363]), but causal evidence is missing and difficult to establish.

## 13 Research Gaps and Future Studies

Despite increasing research efforts during the past decades, our understanding of the main drivers of adiposity in infancy and childhood is still incomplete. In addition to genetic factors and the shared environment of parents with their children, multiple other influencing factors in the postnatal and infancy period require consideration. Moreover, despite considerable epidemiologic evidence for associations between diabetes and obesity of the pregnant women and childhood adiposity risk, causality between the two phenomena still awaits demonstration.

In the above review we have focused mainly on the most likely pathways that connect the intrauterine experience with postnatal factors to explain infant and childhood adiposity. However, multiple questions and research gaps remain. Some of these are summarized below and may hopefully provide a stimulus for future research.

### 13.1 Do the Statistical Associations Reflect Causality?

Since association studies do not establish causality, there is a clear need for RCTs before (e.g., weight loss interventions) or during pregnancy resulting in reduced neonatal adiposity, with suitably long follow‐up of the offspring. This can answer the question of whether changes in adiposity induced in pregnancy track into infancy or childhood by reducing adiposity even despite the shared environment between mothers and offspring. These studies at the juncture of prenatal and postpartum care are notoriously difficult to organize, but engagement with proper stakeholders may facilitate such intervention studies [364].

Alternative methods that may help establish causality include further Mendelian randomization studies, Bayesian network—propensity score matching and structural equation modeling [365]. In combination with RCTs, those studies would give insight into the causal relationship between in utero exposures and childhood adiposity.

### 13.2 How Can Neonatal Adiposity be Predicted?

Although it would be desirable to identify and establish an easily measurable marker of the fetus that predicts neonatal adiposity, fetal growth dynamics with different pregnancy periods having variable effects [163, 366] make this unrealistic. Rather, longitudinal measurements of fetal growth parameters capturing subcutaneous fat deposits with subsequent data modeling of growth velocity [367] may improve our understanding of exposure influences on growth in general and on adipose tissue in particular. These results may be linked with follow up data of adiposity in infancy and childhood.

### 13.3 What Is the Role of Genetics in Determining Adiposity Variability?

Low heritability of adiposity‐related traits suggests a more complex mechanism ultimately determining adiposity variability. However, large‐scale genome‐wide association studies on neonatal and childhood adiposity may change this view. Results of these studies can be used for Mendelian randomization studies allowing causal inference of intrauterine exposures and neonatal or childhood adiposity relationships. Moreover, the results would enable the development of a polygenic risk score to identify individuals at risk for childhood adiposity allowing a targeted intervention before adiposity manifestation. Future studies need to explore gene–gene and gene–environment interactions as plasticity regulators of fetal, neonatal, and childhood adiposity. A potential contribution of genetic robustness that limits phenotypic variation of adiposity traits in the fetus, neonate, and children has remained unclear [16, 368, 369].

### 13.4 What Are the Major Intrauterine Determinants of Adipocyte Number?

As discussed above, triangulation of available evidence may suggest that fetal adipogenesis tracks into the postnatal period and even into childhood and adulthood. Therefore, identifying intrauterine determinants of adipocyte number needs detailed studies. Although challenging in humans, they will provide important information with the vision of devising appropriate therapeutic interventions already during pregnancy. Among others, this requires in‐depth assessment of sex dependency of adipocyte number at all developmental stages and how they are affected by gene–environment interactions.

Oxygen tension variations are major effectors of multiple biological processes outside and within pregnancy [370]. In adults, low oxygen tension affects various cells within the adipose tissue including pre‐adipocytes. Their differentiation to adipocytes is inhibited by low oxygen mediated downregulation of PPAR‐*γ* [371]. Prolonged fetal hyperinsulinaemia as occurs in diabetes or obesity of the pregnant woman can lead to transient oxygen deficit in the fetus [372]. Its potential effect on prenatal adipogenesis warrants investigation as a reduced number of adipocytes in white adipose tissue might limit expansion of white adipose tissue and, hence, its storage capacity [373]. If this occurs in subcutaneous locations, this may lead to a spillover into fat deposition in the viscera.

### 13.5 Adipose Tissue Depot Variation

In adults, the heterogeneity of adipose tissue depots and their effects on health, including risk for Type 2 diabetes and cardiometabolic health, has been well established. While visceral adipose tissue mass is associated with increased risks, subcutaneous adipose tissue mass in the abdomen is unrelated and in the gluteo‐femoral region even associated with a reduced risk [374]. This has led to the concept that not all fat is bad. Indeed, thigh fat may even improve insulin sensitivity [375, 376]. The adipose tissue expandability hypothesis posits that the capacity of individual subcutaneous fat depots to store energy surplus as fat prevents its spillover to the toxic fat deposition around and in organs [377].

In adults, different fat depots respond differently to environmental cues [167, 378]. In normal weight neonates, almost all, that is, 91.5%, of total fat is deposited in subcutaneous depots. Only 8.5% is found in internal depots, of which only about one third is in the intra‐abdominal region [101]. However, it is unclear how conditions such as maternal obesity or diabetes affect fetal fat deposition in various locations. Extreme conditions of metabolic disturbance of the mother are associated with a very modest increase in fetal visceral adipose tissue [166], which may suggest considerable capacity of fetal subcutaneous adipose tissue to expand and store excess fat. A Dutch population‐based cohort study found that fetal growth deceleration, followed by infant growth acceleration was associated with a higher android/gynoid fat mass ratio in children at the age of 6 years [56], suggesting that central adiposity might be stronger influenced by this growth pattern. This is in line with other findings showing that neonates born small with accelerated growth in infancy have more central adiposity between the age of 2 to 4 years [379].

The role of genetic background in determining sensitivity of different fat depots to environmental cues is unclear. It also remains to be studied whether DNA methylation patterns of adipocytes vary between depots in neonates and in childhood.

### 13.6 What Is the Role of Appetite Regulation in Infants?

Leptin is an adipocytokine predominantly produced by and released from adipocytes, with subcutaneous fat depots secreting more leptin than visceral adipose tissue, at least in adults [380]. Its concentrations in cord blood are elevated in pregnancies complicated by diabetes or obesity and associate with neonatal fat mass [231, 381]. This association shows a sex difference, which is accounted for by the different amount of neonatal fat between males and females [231, 232]. Leptin plays a central role in homeostatic regulation of energy balance and metabolism [382]. It regulates appetite and food intake through its action on specific brain centers in the hypothalamus, hippocampus, and brain stem, establishing a negative feedback loop between adipocytes and the brain [383, 384]. Absence or very low serum leptin levels because of leptin gene mutations lead to severe obesity already in childhood [385]. Higher cord blood leptin levels as a result of fetal adiposity may induce central leptin resistance [386] entailing lessened leptin ability to suppress appetite and to increase energy expenditure [383]. This raises the intriguing question of whether leptin resistance acquired in utero can lead to overconsumption of food in the offspring, contributing to adiposity in infancy and childhood [387]. Leptin is present in colostrum and breast milk [388], and higher concentrations are found in breast milk from women with obesity compared to lean women [389, 390]. In breast milk of women with previous GDM, leptin levels are not elevated, probably because of adequate GDM management [391, 392]. In lean mothers, leptin concentrations in breast milk are negatively correlated with infant body weight at 2 years [393]. In contrast to breast milk, leptin is absent from infant formulas [387]. At the age of 4–10 years, leptin concentrations and leptin resistance were higher in children with obesity (defined as BMI ≥ 85th percentile) as compared to those with lower BMI, regardless of maternal obesity or GDM during pregnancy [394].

In addition to leptin, other appetite regulating hormones such as ghrelin [395] need to be studied. Ghrelin is present in cord blood, colostrum, and breast milk [171, 392, 396]. Initial evidence indicates that lower ghrelin concentrations are found in offspring (cord blood and colostrum) and breast milk of women with higher BMI/obesity or GDM [171, 392, 396]. As a decrease in cord blood ghrelin was associated with increased skin fold thickness in offspring at 1 year [171], one may postulate an important role for ghrelin as a contributing factor to infant adiposity, although this needs to be confirmed in other populations. Ultimately, the balance between leptin and ghrelin and other appetite regulating hormones will determine energy homeostasis. If imbalanced, then excessive energy storage and hence adiposity in the offspring will ensue.

In milk of mothers with previous GDM, some metabolite concentrations were altered and few were associated with greater infant weight or length gain in the first 6 months [397]. Future longitudinal studies need to assess which infant body compartment is affected by formula feeding or by breast feeding after pregnancies complicated by diabetes or obesity and how adipocytokines and metabolites are involved.

### 13.7 Further Underappreciated Effect Modifiers

We have previously stressed that perceived psychosocial stress of the pregnant woman, through the activation of stress‐responsive biological systems involving cortisol, among others, and metabolic stress share the same pathway to neonatal adiposity [398] and thus can mutually potentiate each other. Other stressors such as noise exposure, either occupational or traffic noise, can also lead to increased cortisol levels in adults [399] and pregnancy may increase women's sensitivity to noise [400]. In adults, chronic noise exposure is linked to greater adiposity, especially central/visceral fat [401], but there are only a few studies longitudinally associating early noise exposure and other early‐life area‐level characteristics with childhood adiposity [402]. No study assessed potential effects of noise during pregnancy on neonatal adiposity.

Other factors, such as chemical obesogens, for example, endocrine disrupting chemicals, which are capable of influencing adipogenesis and appetite control in adults outside pregnancy [403, 404], may operate in a similar manner in the developing fetus and neonate, but this remains to be demonstrated. Indeed, there is already some evidence of an association of gestational exposure to perfluoralkyl substances with children's adiposity [405, 406], but developmental effects may depend on the specific nature of the endocrine disrupting chemical [407]. Moreover, some effects may be indirect through modification of GDM risk [408] and, hence, risk for neonatal and subsequent childhood adiposity. Environmental pollution with metals may influence childhood adiposity, but the specific effects and pathways are unknown [409, 410]. Air pollution during pregnancy may also affect neonatal and childhood adiposity as particulate matter (PM) with an average diameter of 2.5 nm (PM2.5) can cross the placenta and reach the fetal circulation, where it may affect metabolism, inflammation and other processes ultimately influencing children's health [411, 412]. Some evidence suggests that prenatal PM exposure may lead to increased total fat mass in children at the age of about 8 years [413], calling for more research in this area. However, coherent associations may be difficult to establish because of multiple other confounders, which may mask pollution‐neonatal and childhood adiposity associations and, hence, mask programming effects. Micro‐ and nanoplastics are also well known to affect human health [414], but no studies have assessed their influence on neonatal or childhood adiposity.

Global warming and the associated rising temperatures affect endocrine systems [415], increase the risk of insulin resistance outside [416] and in pregnancy [417] and with it the risk of GDM [418, 419, 420, 421]. The influence of increasing ambient temperature on neonatal adiposity and subsequent development of childhood adiposity has not been studied yet, but temperature effects on white and also brown adipose tissue function warrant these [412, 422].

## 14 Conclusions

While obesity as a categorical diagnosis is an easy to determine trait, it is not useful for understanding pathophysiology and mechanisms of long‐term outcome related to metabolically unhealthy pregnancies. Fat mass, that is, adiposity, is one component determining obesity and the term “adisopathy,” focusing on quality and distribution of fat rather than on its amount, was proposed to redefine obesity [423]. In public health strategies and/or interventions, the measurement of adiposity might be more difficult than obesity, but for understanding mechanisms and estimating long‐term benefits it is necessary to do so. Furthermore, it might be more effective to target people for lifestyle interventions based on adiposity instead of obesity. This would require age and sex specific adiposity cut‐offs for neonates and children, which are missing [423] although body composition growth data are available for various populations and age ranges [107, 424, 425, 426, 427, 428]. Recently, sex‐specific normative data on fat and fat free mass measured by MRI have become available for fetuses from week 30 onward [429].

**FIGURE 5:**
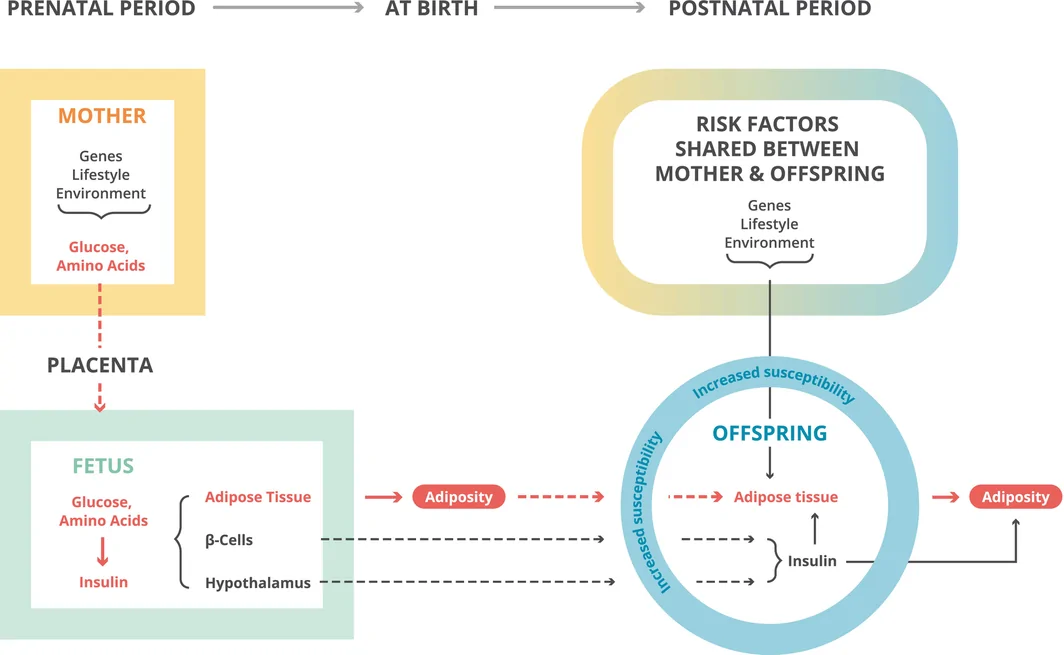
Summary scheme of proposed key pathways leading to childhood adiposity. The pregnant woman (mother) can influence fetal development through her phenotype determined by her genetic background, her lifestyle and her environment. The placenta mediates these influences and passes, among others, insulin secretagogues (glucose, amino acids such as leucine, arginine) from mother to fetus. Whenever the maternal glucose‐insulin axis is disturbed, the resulting hyperglycaemia on the maternal side subsequently leads to fetal hyperinsulinaemia. Insulin is the most important regulator of metabolism and growth and affects a wide array of fetal tissues and organs. It stimulates formation of triglycerides and pre‐adipocyte differentiation to adipocytes capable of storing triglycerides, resulting in fetal adiposity, which at the end of pregnancy precipitates in neonatal adiposity. In the fetal hypothalamus, it can lead to insulin resistance and hence diminished satiety signals, which, if not counteracted by other means, will lead to overfeeding in infancy and beyond. In the pancreas, insulin may stimulate *β*‐cell proliferation. The resulting increased *β*‐cell number will confer the potential to further hyperinsulinemic responses to postnatal challenges. We posit that these changes in key fetal organs sensitize (increase fetal susceptibility in) the offspring for further maternal influences and fetal metabolic changes, foremost of insulin, which continue to contribute to offspring adiposity which can extend into childhood. The effect of these influences is moderated by fetal genetics and the environment shared between mother and fetus and differs between offspring sexes and genders. Insulin continues to be the main driver of adiposity, but other factors acting foremost on cellular constituents of adipose tissue in an autocrine/paracrine manner play a moderating role.

Excess adiposity is not a recently developed problem but has existed since at least the upper Paleolithic [32] allowing evolutionary selection pressures to adapt the physiology of pregnant women and the feto‐placental unit for maternal and fetal protection. Disturbances of the intrauterine environment, in particular metabolic derangements early in pregnancy, such as in women with diabetes and/or obesity, may lead to fetal hyperinsulinaemia. This often results in neonatal adiposity, which may in turn increase the risk for childhood adiposity (Figure 5).

**FIGURE 6:**
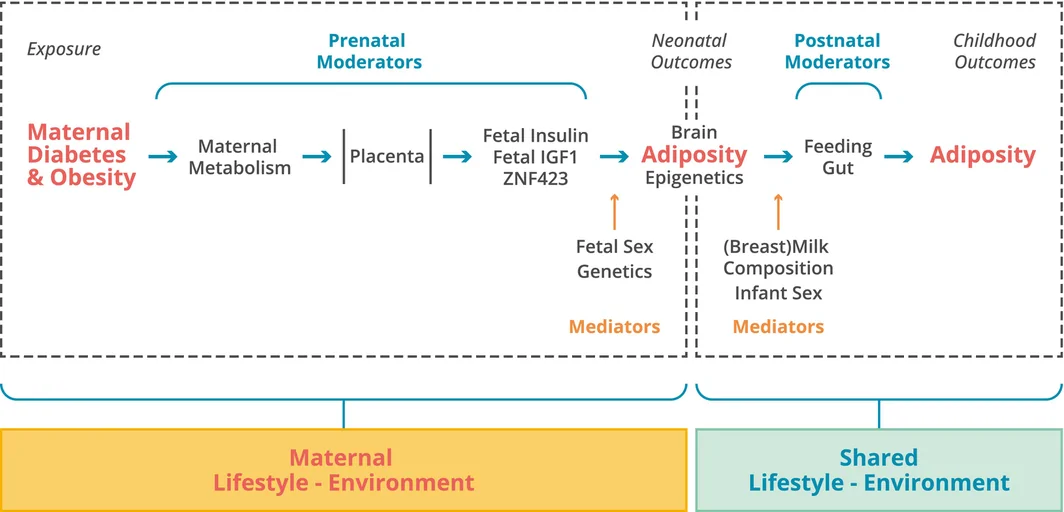
Proposed conceptual framework linking prenatal and postnatal exposures to offspring adiposity. During the prenatal period (left dashed box), maternal diabetes and obesity modify maternal metabolism (moderator), which—potentially via placental adaptations (moderators)—influences fetal circulating insulin and IGF‐1 levels, as well as expression of the transcription factor ZNF423 in fetal adipocytes (moderators). These alterations contribute to the neonatal adipose phenotype, characterized by elevated fetal adiposity and changes in brain development and the fetal epigenome. Fetal sex and genetic make‐up act as mediators of the associations between the intrauterine environment and neonatal outcomes. During the postnatal period (right dashed box), infant feeding influences gut microbiota composition. Milk composition (breastmilk or formula) both moderates and mediates the association between neonatal adiposity and adiposity in later childhood. Infant sex serves as an additional mediator in pathways leading to childhood and potentially adult adiposity. Maternal lifestyle and environmental exposures during pregnancy influence maternal metabolism and, consequently, fetal outcomes, although some exposures may also exert direct effects on the fetus. After birth, the mother and infant share lifestyle and environmental exposures that can modify offspring adiposity trajectories independently of prenatal exposures. This simplified diagram highlights the multifactorial and developmental nature of adiposity in early life. The relative contribution of individual factors may vary across life stages and may be further influenced by currently unknown factors. Only elements discussed in the text are included, and the diagram does not imply causality. Multiple additional moderators and mediators are encompassed within maternal lifestyle and environment during pregnancy and within shared mother–child exposures postnatally. *Moderators* are factors that lie within the pathway between exposure and outcome. *Mediators* influence or modify the association between exposure and outcome but do not lie directly within the pathway. Some factors may function as both moderators and mediators.

Epidemiologic and prospective observational studies have found the association of neonatal fat mass with infancy and childhood adiposity and also suggested an influence of the intrauterine environment on these associations. However, causal evidence in humans is still missing. A variety of postnatal exposures and their interaction with the individual's genetic background contribute to the multifactorial problem of excessive adiposity, which may be further compounded by varying sensitivity to environmental perturbations depending on the developmental stage of the individual [23] (Figure 6).

Based on animal studies, evidence suggests that the in utero environment does influence susceptibility for adverse health outcomes later in life. However, to assume that humans are like mice or rats is false, and based on human data, the jury is still out on fetal programming of adiposity in the deterministic sense as often used.

## Funding

Part of this work was funded by the Austrian Science Fund (FWF) grant number (10.55776/P37199).

## Conflicts of Interest

The authors declare no conflicts of interest.

## Data Availability

No primary data reported here.
